# X-Linked immune dysregulation, polyendocrinopathy, and enteropathy (IPEX) syndrome with concurrent membranous and IgA nephropathy: a case report

**DOI:** 10.1186/s12882-026-05015-8

**Published:** 2026-05-06

**Authors:** Eman Nooreddeen, Abdulrahman Alabdulsalam, Fayhan Alroqi, Ibrahim Sandokji

**Affiliations:** 1 Pediatric Department, Prince Mohammed bin Abdulaziz Hospital, Ministry of National Guard Health Affairs, Madinah, Saudi Arabia; 2 King Abdullah International Medical Research Center (KAIMRC), Riyadh, Saudi Arabia; 3 College of Medicine, King Saud Bin Abdulaziz University for Health Sciences, Riyadh, Saudi Arabia; 4 Department of Pathology and Laboratory Medicine, King Abdulaziz Medical City, Riyadh, Saudi Arabia; 5 Department of Pediatrics, College of Medicine, Taibah University, Madinah, Saudi Arabia; 6 Division of Pediatric Allergy and Immunology, Department of Pediatrics, King Abdullah Specialized Children’s Hospital, King Abdulaziz Medical City, Riyadh, 11426 Saudi Arabia

**Keywords:** Inborn error of immunity, IPEX, Membranous nephropathy, IgA nephropathy, Case report

## Abstract

**Background:**

X-linked immune dysregulation, polyendocrinopathy, and enteropathy (IPEX) syndrome is a rare X-linked disorder caused by mutations in the forkhead box P3 (*FOXP3*) gene. It typically presents very early in life with the classic triad of intractable diarrhea, type 1 diabetes mellitus, and dermatitis. Kidney involvement has been reported in a substantial minority of patients with IPEX syndrome, with membranous nephropathy being the most frequent biopsy pattern.

**Case presentation:**

In this case report, we describe a 15-year-old boy who developed type 1 diabetes mellitus at age one year and later developed difficult-to-control asthma, eczema-like skin rashes, and food allergies. At nine years of age, he was noted to have increasing creatinine levels and proteinuria. Kidney biopsy revealed overlapping features of membranous and immunoglobulin A (IgA) nephropathy. Genetic testing identified a hemizygous pathogenic variant in *FOXP3* (c·1040 G > A, p.Arg347His), establishing a diagnosis of IPEX syndrome. Despite supportive management, his kidney function continued to decline, progressing to stage V chronic kidney disease, and he is now awaiting hematopoietic stem cell transplantation. This case highlights an unusual kidney presentation of IPEX syndrome.

**Conclusions:**

The coexistence of membranous and IgA nephropathy may have contributed to the rapid progression of the disease. Clinicians should consider IPEX syndrome in children with kidney disease accompanied by autoimmune endocrinopathies or allergic features, even if the classic gastrointestinal involvement is missing.

## Background

X-linked immune dysregulation, polyendocrinopathy, and enteropathy (IPEX) syndrome was first described by Powell et al. in the early 1980s [1]. It is a rare genetic disorder caused by mutation in the forkhead box P3 (*FOXP3*) gene, located on the X chromosome, that primarily affects the function of thymic-origin regulatory T cells, leading to autoimmunity, immune deficiency, and allergies [2]. Since its initial description, cases have been increasingly recognized over the years, reflecting an increasing awareness of IPEX syndrome in the medical community [3]. Clear genotype-phenotype correlations remain limited among patients diagnosed with IPEX syndrome, with significant heterogeneity observed across reported cohorts [4, 5]. Severe forms of IPEX syndrome are associated with devastating outcomes, often resulting in death if left untreated [6]. IPEX syndrome can even occur during fetal development, resulting in miscarriage or hydrops [7, 8]. Most surviving neonates and infants present during their first year of life [2, 6].

The classic presentation of IPEX syndrome comprises a triad of symptoms: diarrhea in nearly all cases (97.7%), often intractable; type 1 diabetes mellitus, which is the most common endocrinopathy developing after birth or within the first year of life in most cases; and dermatitis (62%) [2, 4, 5]. Kidney involvement has been reported in a substantial minority of patients and is usually identified early in life. Membranous nephropathy is the most common finding in kidney biopsies [9]. Here, we present the first known case of IPEX syndrome with a pathogenic *FOXP3* mutation and kidney biopsy consistent with both membranous and IgA nephropathy, expanding the recognized renal spectrum of this rare disorder.

### Case presentation

This is a 15-year-old boy who had been diagnosed with type 1 diabetes mellitus at the age of one year, and his hemoglobin A1c (HgbA1c) level had been consistently between 8 and 9%. He experienced unexplained failure to thrive after his first year of life and suffers from bronchial asthma. He is also known to have an immunoglobulin E-mediated food allergy to nuts and bananas.

At nine years of age, the patient’s serum creatinine level increased from a baseline of 50 to 100 μmol/, which was persistent despite fluid challenge. The estimated glomerular filtration rate (eGFR) using the CKiD U25 formula was 42.1 mL/min/1.73 m^2^ (CKD stage IIIb). He had no peripheral edema, and his blood pressure was 101/69 mmHg (<90th percentile for age, height, and sex). Urinalysis revealed protein +1 to +2, no microscopic hematuria, no dysmorphic red blood cells, and no cellular casts identified. The 24-hour urine protein excretion was 0.64 g/day, but was inaccurate, while the random urine sample protein to creatinine ratio was 2.4 mg/mg, and his serum albumin was 35 g/L. Additional basic laboratory tests are summarized in Table 1. Ultrasound showed small kidneys measuring up to 7 cm in maximum length which falls below the 2.5^th^ percentile for height (while the expected 50^th^ percentile for his height is 8.5 cm), increased parenchymal echogenicity, and poor corticomedullary differentiation. He had further investigations that included immunological and serological work-up that were negative and are summarized in Table 2. His parents declined the needed kidney biopsy at that time. He was managed for the proteinuria by anti-proteinuric agent, and sodium bicarbonate supplementation was started. Several months later, the patient’s serum creatinine level reached 200 μmol/L, the 24-hour urine protein excretion was 1.3 g/day, urine protein to creatinine ratio was 2.6 mg/mg, serum albumin was 40 g/L, and he continued to have no edema, and normal blood pressure. No immunosuppressive therapy was initiated during this period as the parents initially declined kidney biopsy, and the etiology of kidney disease remained unclear.

**Table 1:** The patient’s laboratory work-up at the time of initial presentation with kidney dysfunction at age 9 years (Result 1) and after one year at the time of kidney biopsy (Result 2)

Test	Result 1	Result 2	Lab Reference Range
**Serum Chemistry**			
Total Protein (g/L)	61	65	60–80
Albumin (g/L)	35	40	35–50
Uric Acid (μmol/L)	150	226	180–340
BUN (mmol/L)	6.2	9.7	2.5–7.1
Creatinine (μmol/L)	100	200	44–88
Sodium (mmol/L)	140	139	136–145
Potassium (mmol/L)	3.7	3.8	3.5–5.1
Chloride (mmol/L)	113	112	98–107
Bicarbonate (mmol/L)	18	19	22–29
Adjusted Calcium (mmol/L)	2.36	2.21	2.2–2.7
Phosphate (mmol/L)	1.14	1.24	1.0–1.8
PTH (pg/ml)	46	91	15–68
**Urinalysis and urine protein measurement**			
Urine RBC/HPF	0–2	0–2	0–5
Urine WBC/HPF	0–2	0–2	0–5
Urine protein to creatinine ratio (mg/mg)	2.4	2.6	<0.2

BU, blood urea nitrogen; PTH, parathyroid hormone, RBC, red blood cell; WBC, white blood cell; HPF, high power field

**Table 2:** The patient’s immunological and serological work-up

Test	Result	Reference Range	Interpretation
**Autoantibodies**			
ANA	Negative	Negative	Negative
Anti-dsDNA (IU/mL)	90.71	0–200	Negative
c-ANCA (U)	0.25	≤20	Negative
p-ANCA (U)	2.72	≤20	Negative
Anti-GBM (U)	3.8	≤20	Negative
Anti-TTG IgG (U)	8.04	<20	Negative
Anti-TTG IgA (U)	2.89	<20	Negative
Thyroid Peroxidase Antibodies (IU/mL)	<1	1–16	Negative
Thyroglobulin Antibodies (IU/mL)	6.26	5–100	Negative
PLA2R1 (titer)	<1:10	<1:10	Negative
THSD7A (titer)	<1:10	<1:10	Negative
**Viral Serologies**			
HBV surface Ag	Nonreactive	-	Negative
Anti-HBsAg (mIU/mL)	3.7	-	Non-immune
Anti-HBc	Nonreactive	-	Negative
HCV antibody	Nonreactive	-	Negative
HAV IgM	Nonreactive	-	Negative
HAV IgG	Nonreactive	-	Negative
HIV Ag/Ab	Nonreactive	-	Negative
CMV IgG (U/mL)	176	<14	Positive
CMV IgM	Negative	-	Negative
EBV IgG (U/mL)	80.3	<20	Positive
EBV IgM	Negative	-	Negative
HSV I & II IgG	Positive	-	Positive
HSV I & II IgM	Negative	-	Negative
Mumps IgG (AU/mL)	29.3	<11	Positive
Measles IgG (AU/mL)	<5	<13.5	Negative
Tetanus toxoid IgG (IU/mL)	0.08	<0.1	Non-protective
**Immunoglobulins**			
Total IgE (kU/L)	221	2–629	Normal
Total IgG (g/L)	10.20	7.5–15.6	Normal
Total IgM (g/L)	1.24	0.46–3.04	Normal
Total IgA (g/L)	2.00	0.82–4.53	Normal
**Others**			
C3 (g/L)	1.15	0.9–1.9	Normal
C4 (g/L)	0.31	0.1–0.4	Normal

ANA, anti-nuclear antibodies; ANCA, anti-neutrophil cytoplasmic antibodies; anti-dsDNA, double-stranded DNA antibodies; anti-GBM, anti-glomerular basement membrane antibodies; anti-HBV core, anti-hepatitis B virus core antibodies; anti-HBVsAg, anti-hepatitis B virus surface antigen antibodies; anti-TTG, anti-tissue transglutaminase antibodies; C3, complement 3; C4, complement 4; CMV, cytomegalovirus; EBV, Epstein–Barr virus; HAV, hepatitis A virus; HBV surface Ag, hepatitis B virus surface antigen; HCV, hepatitis C virus; HIV, human immunodeficiency virus; HSV, herpes simplex virus; IgA, immunoglobulin A; IgE, immunoglobulin E; IgG, immunoglobulin G; IgM, immunoglobulin M; PLA2R, phospholipase A2 receptor; THSD7A, thrombospondin type 1 domain containing 7A. PTH, parathyroid hormone

**Fig. 1:**
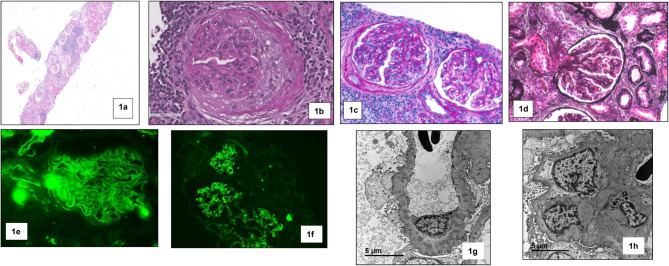
Kidney biopsy findings demonstrating coexisting membranous nephropathy and IgA nephropathy. (**a**) Marked tubulointerstitial scarring (H&E stain, 40x). (**b**) Fibrocellular crescent (PAS stain, 400×). (**c**) Diffusely thickened glomerular capillary loops and mild mesangial expansion (PAS stain, 400×). (**d**) Segmental holes and spike formation along the capillary loops (Jones silver stain, 400×). (**e**) Fine granular IgG deposits along the glomerular capillary walls (immunofluorescence). (**f**) Strong smudgy IgA deposits in the mesangium (immunofluorescence). (**g**) Subepithelial and intramembranous electron-dense deposits (electron microscopy). (**h**) Mesangial electron-dense deposits (electron microscopy)

Kidney biopsy was eventually performed at age 10 years after obtaining parent consent. The biopsy specimen contained 33 glomeruli, of which 9 (27%) showed global sclerosis (Fig. 1). Light microscopy revealed glomerular segmental sclerosis in two glomeruli, mesangial proliferation, capillary wall thickening, and focal fibrocellular crescents, set against a background of significant tubular atrophy and interstitial fibrosis (estimated at 50% of cortical area) (Fig. 1). Moderate interstitial inflammation predominantly composed of lymphocytes was present and there was no evidence of vasculitis. Immunofluorescence study showed two distinct patterns: the first is a smudgy mesangial staining, positive for immunoglobulin A (IgA, +3), and Lambda light chains (+2), and the second is fine granular glomerular capillary staining for immunoglobulin G (IgG, 2+), immunoglobulin M (IgM, +1), complement C3 (C3, 2+), and Kappa light chains (2+) (Fig. 1). Staining for complement component 1q (C1q) and complement 4 (C4) was negative. Ultrastructural examination confirmed immune-type electron-dense deposits in mesangium and glomerular basement membranes, specifically on subepithelial and intramembranous regions (Fig. 1). The overall findings were interpreted as the coexistence of IgA nephropathy (M1 E0 S1 T2 C1, according to the Oxford classification) with membranous nephropathy (features most consistent with stage II-III based on ultrastructural findings) [10]. No definitive histological features of diabetic nephropathy (such as nodular mesangial sclerosis or arteriolar hyalinosis) were identified on the biopsy specimen, though the severe chronic changes may have obscured subtle diabetic changes. Staining for IgG subclasses and phospholipase A2 receptor 1 (PLA2R1) was unavailable. The known autoantibodies involved in primary membranous nephropathy including PLA2R1) and additional other serologies were negative (Table 2).

After the findings of the kidney biopsy and the further work-up, the patient continued the anti-proteinuric agent, and the sodium bicarbonate supplementation. Optimizing diabetes mellitus control was recommended, but his HgbA1c continued in the range between 8 and 9%. The proteinuria showed initial response in the urine protein to creatinine ratio reaching 0.2 mg/mg. However, this response was only for a short period after which the urine protein to creatinine ratio was persistent in the range of 1.9–2.9 mg/mg with normal serum albumin level and no edema, despite compliance to the prescribed anti-proteinuric agent. During follow up, treatments were added for the arising CKD complications. Alfacalcidol and calcium supplementation were added to manage secondary hyperparathyroidism. Later when the patient developed hypertension, calcium channel blocker was added as second agent for hypertension control. Moreover, his CKD continued to progress over the years of follow up. Human growth hormone therapy was started for growth retardation. No immunosuppression was started as the underlying etiology of his kidney biopsy findings was unclear.

**Fig. 2:**
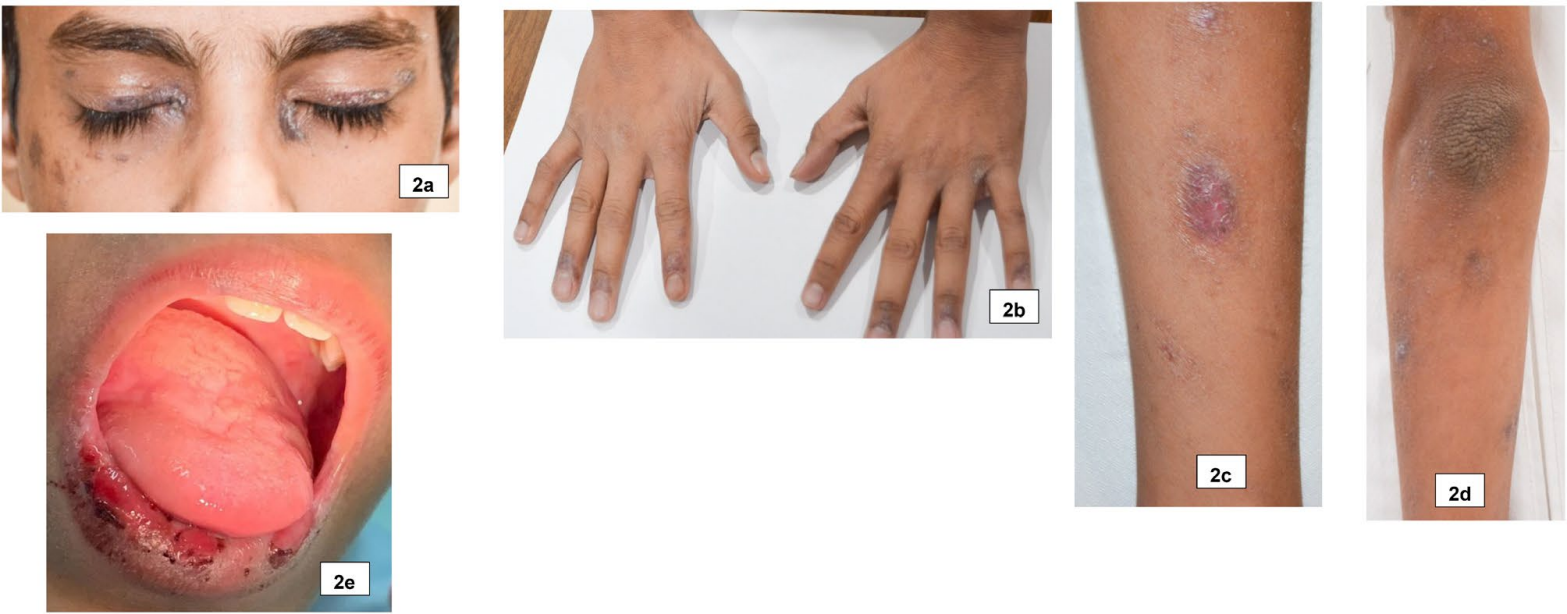
The patient’s clinical features, including eczema-like lesions affecting his (**a**) eyelids, (**b**) hands, upper limb (**c**) ventral and (**e**) dorsal sides, and (**e**) a persistent ulcer on his lower lip, which were diagnosed as lichen planus

At age 11 years, he developed eczema-like lesions, mainly affecting the hands, upper limbs, and later the eyelids (Fig. 2) which responded to topical steroids but recurred frequently. Subsequently, at age 13 years, he started to have a non-healing ulcer on his lower lip and a geographic tongue that lasted for a few months and did not respond to a course of intravenous acyclovir (Fig. 2). He was then diagnosed with skin and lip lesions consistent with lichen planus and was started on topical 1% pimecrolimus cream, to which he responded well. The combination of early-onset type 1 diabetes mellitus, severe allergic manifestations, progressive kidney disease, recurrent dermatological lesions, and failure to thrive ultimately prompted consideration of an underlying inborn error of immunity with immune dysregulation. Whole exome sequencing (WES) was performed as described previously in our earlier report [11]. The result of the genetic study revealed a hemizygous variant in *FOXP3* (c.1040 G > A, p. Arg347His) that was confirmed by Sanger sequencing. The identified variant is located in the forkhead DNA binding domain, a critical functional domain of the FOXP3 protein and has been previously reported in patients with IPEX syndrome with defective regulatory T cell function. This variant is classified as pathogenic according to the American College of Medical Genetics and Genomics (ACMG) guidelines. His parents are first cousins; however, none of his nine older siblings are symptomatic. His family history revealed no similar conditions including kidney disease, or immune deficiency. Detailed humoral immune profiling, including autoantibodies, immunoglobulins, and specific antibody titers, is presented in Table 2. Lymphocyte subset analysis showed elevated levels of memory T cells and reduced levels of regulatory T cells. The peripheral eosinophil counts over the years ranged from 0.19 to 1.79 × 10^9^/L, representing 5%-16% of the total white blood cell count. The liver and thyroid function tests were normal.

**Fig. 3:**
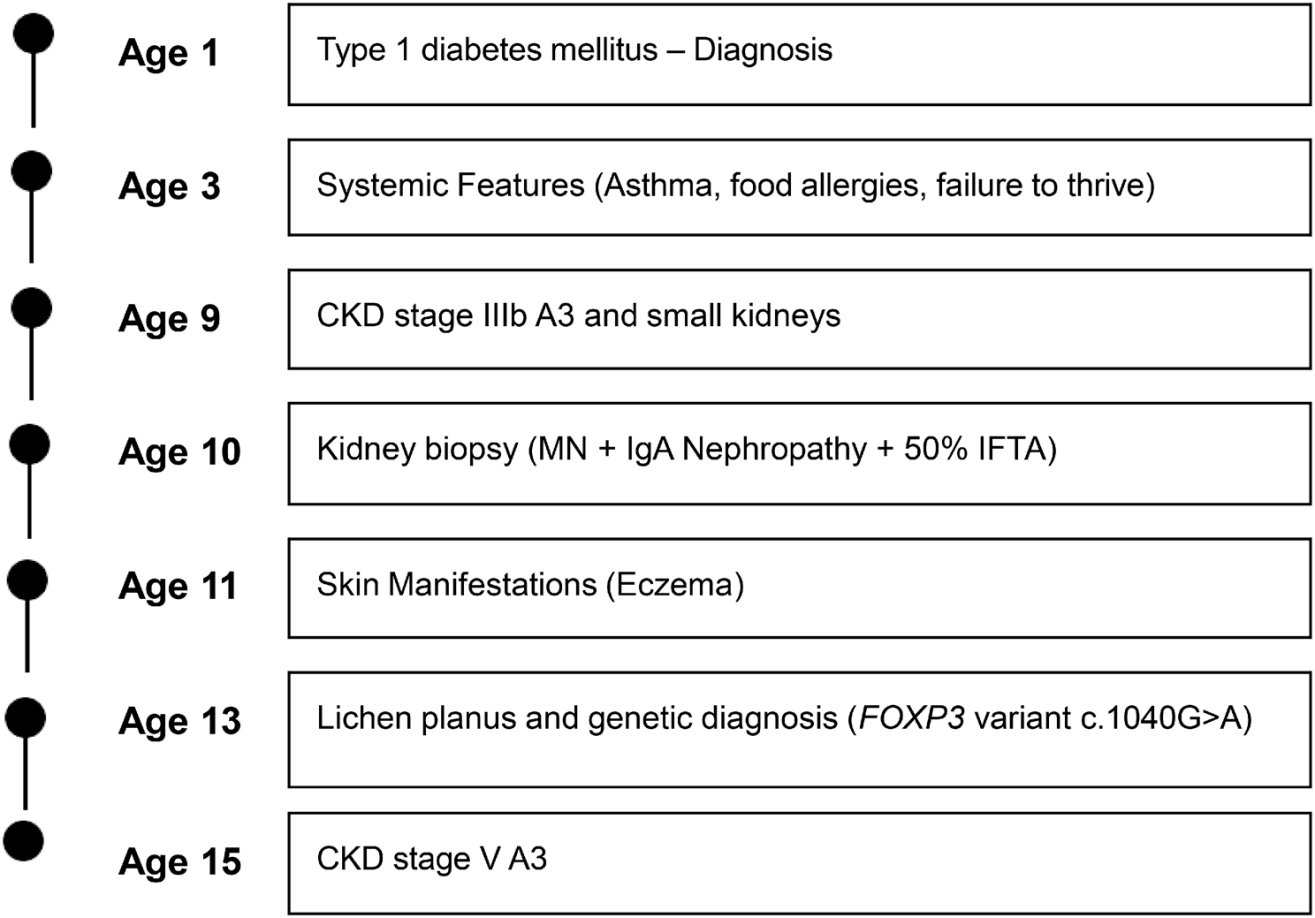
Clinical timeline diagram for this IPEX syndrome case report. Abbreviation definition: CKD, chronic kidney disease; stage, classification by CKiD U25 equation; A, proteinuria by urine protein to creatinine ratio; MN, membranous nephropathy; IFTA, interstitial fibrosis and tubular atrophy

He is currently 15 years old, with CKD stage V A3, but has not yet started dialysis. His latest serum creatinine level is 455 μmol/L, urine protein to creatinine ratio is 3.28 mg/mg, urine albumin to creatinine ratio is 79 mg/mmol, and serum albumin is 39 g/L. He is currently awaiting hematopoietic stem cell transplantation (HSCT). The clinical timeline of patient’s presentation is summarized in Fig. 3.

## Discussion

To our knowledge, based on a systematic search of PubMed, Embase, and Google Scholar databases (search terms: “IPEX syndrome,” “FOXP3,” “IgA nephropathy,” “membranous nephropathy,” “renal involvement”; last searched: February 2026), this is the first reported case of IPEX syndrome with biopsy-proven concurrent membranous and IgA nephropathy. While membranous nephropathy has been well-documented in IPEX syndrome, and isolated IgA deposits have occasionally been mentioned, the coexistence of both pathologies with the characteristic histological features of IgA nephropathy (Oxford classification M1 E0 S1 T2 C1) and membranous nephropathy has not been previously described. Unlike previously reported cases, our case developed both membranous and IgA nephropathy at an older age without enteropathy.

Most reported patients with IPEX syndrome and kidney involvement who underwent kidney biopsy were younger, except for one who was 11 years old [9]. Histopathological findings in kidney biopsies often reveal membranous nephropathy. However, other conditions have also been reported, including interstitial nephritis, tubulointerstitial nephritis, minimal change disease, and membranoproliferative glomerulonephritis [9]. Immunofluorescence microscopy of kidney tissue typically shows the deposition of IgG, C3, complement component 1q (C1q), and a split product of C4 (C4d) [9]. Our patient exhibited features of both membranous and IgA nephropathy in the kidney biopsy, against a background of severe interstitial fibrosis and tubular atrophy. An important consideration is whether the IgA deposits represent true IgA nephropathy or incidental IgA deposition. IgA nephropathy is relatively common in the general population (particularly in Asian populations), and IgA deposits can occasionally be found in glomeruli affected by other primary diseases or in sclerotic glomeruli as a “trapping” phenomenon. However, several features in our case support a diagnosis of true IgA nephropathy coexisting with membranous nephropathy [1]: the intense (3+) mesangial IgA staining with co-dominant Lambda light chains [2]; the presence of mesangial proliferation and crescents on light microscopy [3]; electron-dense deposits in the mesangium on electron microscopy [4]; the Oxford classification features (M1 E0 S1 T2 C1) indicating active IgA nephropathy with significant chronicity; and [5] the immunofluorescence results were only interpreted on non-sclerotic glomeruli which makes the IgA deposition related to “trapping” phenomenon unlikely. Nevertheless, we acknowledge that the relative contribution of each pathology to the overall kidney dysfunction and progression is difficult to determine, especially given the severe underlying chronic changes.

The differential diagnoses for a child presenting with type 1 diabetes mellitus, kidney disease with combined membranous and IgA nephropathy, and dermatological manifestations were studied in our patient. Systemic lupus erythematosus (SLE) was considered, but the patient did not meet the diagnostic criteria according to either the 2019 EULAR/ACR classification criteria or the 1997 ACR criteria. The anti-nuclear antibodies (ANA) and anti-double-stranded DNA (anti-dsDNA) were negative, and serum complement 3 & 4 levels were normal (Table 2). The patient lacked other clinical features of SLE such as malar rash, photosensitivity, serositis, arthritis, and neurological manifestations. The eczema-like rash and lichen planus were more consistent with the dermatological manifestations of immune dysregulation syndromes. Infection related-IgA nephropathy was less likely because the patient was lacking the clinical features of hypertension and edema at the initial presentation, and there was no hematuria before the kidney biopsy. He also had no history of current or recent infections. Serological investigations were performed to evaluate for primary membranous nephropathy including anti-phospholipase A2 receptor (PLA2R1) antibody and anti-thrombospondin type 1 domain-containing 7A (THSD7A) antibody and were negative. Secondary membranous nephropathy was evaluated through testing for hepatitis B, hepatitis C, and HIV and all were negative (Table 2). Other considerations included diabetic nephropathy, though the patient was relatively young and the histology was not typical (i.e. among the known features of diabetic glomerulopathy, there is only mild mesangial matrix expansion, which is not specific to diabetic nephropathy). Anti-neutrophil cytoplasmic antibodies (c-ANCA and p-ANCA) and anti-glomerular basement membrane (anti-GBM) antibodies were negative, excluding ANCA-associated vasculitis and anti-GBM disease. After considering all the workup that was done, in addition to the unusual kidney histological pattern with the associated systemic features, suspicion was raised for an underlying genetic disorder affecting immune regulation.

The rapid progression of CKD in our patient warrants careful consideration of contributing factors. While the coexistence of membranous and IgA nephropathy likely played a role, the finding of small kidneys (7 cm at age 9 years, below the 3^rd^ percentile), poor corticomedullary differentiation on ultrasound could be related to an underlying kidney hypodysplasia or pre-existing chronic kidney damage that may have been significant contributors to the kidney function decline. Proteinuria is considered another significant factor of CKD progression. Additionally, the sub-optimal control of type 1 diabetes mellitus (HbA1c consistently 8–9%) may have contributed to diabetic nephropathy, though specific histological features of diabetic nephropathy were not prominent on biopsy. The severe chronic changes observed at the time of biopsy (27% global glomerulosclerosis, 50% interstitial fibrosis) likely explain a substantial part of the subsequent deterioration in kidney function. Therefore, the progression to stage V CKD over the follow up period of 6 years likely reflects a multifactorial process involving glomerular disease (both membranous and IgA nephropathy), possible underlying structural changes (kidney hypodysplasia), proteinuria, and possible metabolic injury from diabetes.

The decision not to pursue immunosuppressive therapy requires explanation. At the time of kidney biopsy (age 10 years), the genetic diagnosis had not yet been established, and the etiology of the combined membranous and IgA nephropathy was unclear. By the time IPEX syndrome was diagnosed at age 13 years, the patient had already progressed to CKD stage IV (by CKiD U25 equation using serum creatinine) with severe chronic changes on the initial biopsy (50% interstitial fibrosis and tubular atrophy). At this advanced stage of CKD, the potential risks of immunosuppression (infection, malignancy, drug toxicity) were felt to outweigh potential benefits, as the severe chronic damage was considered largely irreversible. Regarding mammalian target of rapamycin (mTOR) inhibitors, sirolimus was discussed as a potential bridging therapy prior to HSCT, given its dual benefits of immunosuppression and antiproliferative effects. However, concerns about impaired wound healing, potential exacerbation of proteinuria, and the patient’s already compromised kidney function led to the decision to proceed directly to HSCT evaluation without interim immunosuppression. In retrospect, diagnosis of IPEX and initiation of sirolimus or other immunosuppression at an earlier stage of CKD might have slowed disease progression. Conservative management for proteinuric CKD was optimized within the constraints of progressive kidney dysfunction. Blood pressure was controlled with amlodipine and angiotensin receptor blocker. The patient received dietary counseling for CKD-appropriate nutrition. Diabetes management was intensified, though HbA1c control remained suboptimal.

Atypical presentations of IPEX syndrome have been reported, including older age at onset, the absence of the triad of organ involvement, and milder phenotypes, causing challenges and delays in its diagnosis [12, 13]. Our patient experienced a delayed diagnosis of IPEX syndrome due to its rarity, as well as his atypical features, such as the lack of gastrointestinal involvement and a delayed diagnosis of type 1 diabetes mellitus after infancy. Given our patient’s advanced CKD, immunosuppression was infeasible, highlighting the importance of early recognition and referral for HSCT. Current treatments for IPEX syndrome include HSCT and immunosuppression with mTOR or calcineurin inhibitors. However, a risk of mortality remains, especially in the first 2.5 years after HSCT, or later, when more organs are affected following immunosuppression [2–5]. The survival rate depends on the number of organs involved before HSCT [3]. A multicenter retrospective study of 96 patients with IPEX syndrome found that 10 underwent HSCT after the age of 10 years; however, the follow-up period for these patients was insufficient to provide insights into their long-term prognosis [3]. Therefore, predicting the outcome after HSCT for our patient is challenging, especially since he was diagnosed late and has three affected organs (pancreas, kidney, and skin). Gene-based therapy shows promise but is complex and requires further evaluation for patients with IPEX syndrome [14]. Genetic counseling is crucial in these cases to prevent disease recurrence in future pregnancies.

## Conclusion

Our patient with IPEX syndrome, who has both membranous and IgA nephropathy in kidney tissue biopsy, is to our knowledge the first reported case in the literature to demonstrate this combination of histopathological findings. Due to the variety of phenotypes and the increasing number of patients with milder and atypical presentations, IPEX syndrome should be considered in the differential diagnosis of any child with kidney disease associated with other autoimmune disease(s) or allergic manifestations, especially when endocrinopathy is present, even in the absence of classic gastrointestinal features.

### Limitations

Our case report has several limitations that should be acknowledged. First, IgG subclass staining was not performed on the kidney biopsy specimen, which would have provided additional information about the nature of the membranous nephropathy (IgG4-dominant staining is typical of primary membranous nephropathy, while other IgG subclasses suggest secondary forms). Second, although PLA2R1 and THSD7A antibodies were tested and were negative, other emerging membranous nephropathy-associated antigens (such as NELL1, semaphorin 3B, and others) were not evaluated due to limited availability. Third, staining for additional complement components (C4d, C5b-9) was not performed due to tissue limitations. Fourth, the retrospective nature of this case report means that some clinical details and serial measurements were not as comprehensive as would be ideal in a prospective study.

## Data Availability

The original contributions in this study are included in the article; for further inquiries, please contact the corresponding author.

## Abbreviations

IPEX: X-Linked Immune DysregulationPolyendocrinopathy, and Enteropathy
*FOXP3*: Forkhead box P3 gene
IgG: Immunoglobulin G
C3: Complement 3
IgA: Immunoglobulin A
PLA2R1: Phospholipase A2 receptor 1
C1q: Complement component 1q
C4d: Split product of C4
CKD: Chronic kidney disease
WES: Whole exome sequencing
HSCT: Hematopoietic stem cell transplantation
mTOR: Mammalian target of rapamycin
